# PSYCHOLOGICAL RESPONSES TO IMPENDING AND PAST LOSS OF A LOVED ONE

**DOI:** 10.1093/geroni/igac059.245

**Published:** 2022-12-20

**Authors:** Holly Prigerson

**Affiliations:** Weill Cornell Medicine, New York City, New York, United States

## Abstract

Psychological responses to an impending or recent death have received minimal attention in the research literature. I will present data to demonstrate the high levels of psychological distress (e.g. symptoms of peritraumatic stress, anxiety, depression and grief) reported by caregivers of a loved one with a life-threatening illness. I will report on studies that have examined how grief symptoms change over time, or not, as in the case of symptoms of Prolonged Grief Disorder (PGD) – a new addition to the Diagnostic and Statistical Manual of Mental Disorders -5-Text Revision (DSM-5-TR). A case will then be made for addressing psychological struggles of caregivers of patients who are dying in the intensive care unit (ICU). I will then present a conceptual model and manual of our psychosocial intervention (EMPOWER), which we designed to reduce symptoms of PGD and posttraumatic stress disorder (PTSD) by targeting symptoms of experiential avoidance and promoting adaptive coping.

